# Understanding Public Emotions: Spatiotemporal Dynamics in the Post-Pandemic Era Through Weibo Data

**DOI:** 10.3390/bs15030364

**Published:** 2025-03-14

**Authors:** Yi Liu, Xiaohan Yan, Tiezhong Liu, Yan Chen

**Affiliations:** 1 School of Management, Beijing Institute of Technology, Beijing 100081, China; liuyisme1996@bit.edu.cn (Y.L.); yxh0522@bit.edu.cn (X.Y.); liutiezhong@bit.edu.cn (T.L.); 2 Crisis Management Research Center, Beijing Institute of Technology, Beijing 100081, China

**Keywords:** spatiotemporal characteristics, public emotion, post-pandemic era, spatial autocorrelation, social media, China

## Abstract

Prolonged exposure to public health crises in the post-pandemic era poses significant threats to global mental health. To address this, we developed a conceptual model to analyse the spatiotemporal distribution of public emotions, using Weibo data from the 2022 Beijing bar outbreak (9 June–18 August). The model integrates lexicon-based emotion analysis, spatial autocorrelation tests, and content analysis to provide a comprehensive understanding of emotional responses across stages and regions. The findings reveal a multi-peak emotional cycle spanning emergency, contagion, and resolution stages, with significant emotional clustering in emergency zones, surrounding areas, and regions visited by infected individuals. Through coding, we identified 24 main-categories and 90 sub-categories, distilled into nine core themes that illustrate the interplay between influencing factors, public emotions, and online behaviours. Positive public emotions (e.g., hopefulness, gratitude, optimism) were linked to pandemic improvements and policy implementation, driving behaviours such as supporting prevention measures and resisting misinformation. Negative emotions (e.g., anger, anxiety, sadness) stemmed from severe outbreaks, insufficient controls, and restrictions on freedoms, leading to criticism and calls for accountability. This study bridges big data analytics with behavioural science, offering critical insights into evolving public emotions and behaviours. By highlighting spatiotemporal patterns and emotional dynamics, it provides actionable guidance for governments and health organizations to design targeted interventions, foster resilience, and better manage future social crises with precision and empathy.

## 1. Introduction

The coronavirus disease 2019 (COVID-19) pandemic is a multifaceted crisis and a potentially traumatic event with profound implications for mental health. Existing research on major public health emergencies has shown that, following the peak of a pandemic, both general populations and vulnerable groups may experience a range of mental health issues (Vadivel et al., 2021). These include sadness, anxiety, depression, panic, post-traumatic stress disorder (PTSD), and suicidality (Das, 2020), as well as stigma, discrimination, and collective behaviours associated with the pandemic (Ransing et al., 2020). Moreover, outbreaks in the post-pandemic era have highlighted the interconnectedness of public health crises and the dynamics of public emotions. These emotions often exhibit a synergistic effect during crises, with similar events evoking comparable emotional responses (An et al., 2022), potentially leading to social polarisation and affecting societal stability and security. Online social media platforms play a critical role in shaping and amplifying public emotions during public health crises. They serve as “amplifiers” and “virtual sensors” of public opinion, enabling the rapid dissemination of emotions across temporal and spatial boundaries (Zhang & Cheng, 2021; He et al., 2024). The convenience of information dissemination on these platforms has made them indispensable for public communication and emotional expression (Yuan et al., 2021; Fang et al., 2022), especially during crises (Mosleh et al., 2021). The rapid increase in public interaction and communication on social media networks during the pandemic has had a profound impact on public emotions (Y. Li et al., 2019; Islm et al., 2021; Ahmad & Murad, 2020), underscoring the platform’s role in emotional dynamics during emergencies. In the context of post-pandemic public health crises, analysing residents’ emotions expressed on social media and understanding their spatiotemporal evolutionary characteristics is essential. Such analysis not only aids in mitigating extreme public emotions and reducing collective behaviours but also offers a novel approach to managing public emotions during health crises, thereby contributing to enhanced societal resilience and stability.

Research leveraging social media data to study public emotions has primarily focused on the temporal characteristics and thematic evolution of emotional responses. For instance, studies have examined the temporal dynamics and thematic shifts of public emotions among different social media users during emergencies (Gu et al., 2021; Naskar et al., 2020), as well as the evolution of public emotions and topics during public health crises such as the COVID-19 pandemic in China (Q. Li et al., 2020). Beyond this, the analytical focus has gradually expanded from a single temporal dimension to encompass spatial features. This includes investigations into how spatial location influences public emotions during the COVID-19 pandemic (Shen et al., 2021). In recent years, the integration of temporal and spatial dimensions has facilitated a deeper understanding of the spatiotemporal evolution of public emotions during emergencies. Research in this domain has explored spatiotemporal changes in emotions associated with major events (Xiao et al., 2022), examined the dynamics of social emotions to understand the emergence of social movements (Tang, 2021), and analysed the classification and spatiotemporal characteristics of emotions during the COVID-19 pandemic (T. Li et al., 2022; Zhu et al., 2020). These approaches have highlighted the importance of coupling spatiotemporal dimensions for a more comprehensive analysis of public emotion evolution. In addition to these quantitative methodologies, qualitative approaches have also been employed. For example, qualitative content analysis has been applied to emotion analysis, with studies manually coding social media content to assess emotional dynamics (S. J. Guo, 2017), such as during the first COVID-19 lockdown in Wuhan, China (Y. Guo et al., 2023). These qualitative methods complement quantitative analyses, offering nuanced insights into the validity and context of emotional expressions in social media data. By integrating spatiotemporal and thematic dimensions, these studies provide a foundation for understanding the evolution of public emotions during crises, demonstrating the value of geo-information methods in public health research.

In summary, current spatiotemporal analyses of public emotions face significant limitations. One key issue is the independent treatment of spatiotemporal dimensions, often neglecting the synergistic interplay between spatial and temporal trends. Additionally, while most existing studies focus on public emotions during the pandemic, they pay insufficient attention to the post-pandemic phase-a period with a heightened risk of psychological challenges. To address these gaps, this study examines the June 2022 bar outbreak in Beijing as an illustrative case of the post-pandemic era. The outbreak originated in a well-known bar where many patrons were not required to wear masks or provide proof of a negative nucleic acid test (NAT). Close social interactions in a crowded indoor setting further accelerated the virus’s spread, triggering a citywide surge in cases. Using data from Weibo, a widely used social media platform in China, this study constructs a highly relevant dataset. Grounded in the public opinion evolution cycle, it employs a range of analytical methods to examine the spatiotemporal dynamics of public emotions and discussion topics across different periods. By integrating these dimensions, the study provides a comprehensive analysis of how public emotions evolve over time and space, offering deeper insights into their geographical distribution and patterns.

Our research focuses on four key questions:

(1) What is the cycle of the evolution of public emotions in the post-pandemic era?
(2) Is there spatial heterogeneity in public emotions during the post-pandemic era? How does emotion vary across regions?
(3) What are the differences in public concern topics under polarised emotions, and what factors influence these emotions?
(4) What are the spatiotemporal patterns in the distribution of public focus on themes?

The findings of this study have practical implications for future research and public emotion governance in the post-pandemic era. By addressing the dynamic shifts in public sentiment and their spatial distributions, the study offers valuable guidance for managing emotional dynamics during public health crises. Furthermore, it contributes to the theoretical understanding of the transition of social order from instability at the pandemic’s peak to equilibrium during the post-pandemic period. This research underscores the importance of integrating geospatial methodologies and public emotion analysis for effective crisis management and societal resilience in the face of future health emergencies.

## 2. Data and Methods

### 2.1. Study Case Description

**Figure 1:**
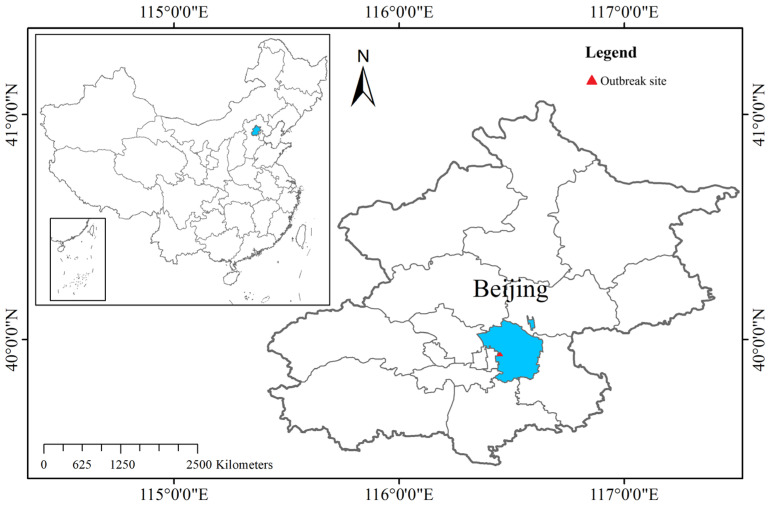
Location of Chaoyang district, Beijing and outbreak site.

Beijing (115°20′–117°30′ E, 39°28′–41°05′ N), located in northern China (Figure 1), is the nation’s capital and a mega first-tier metropolis. Covering a total area of 16,410 square kilometres, it features a complex urban structure and a highly dynamic population, reflecting its status as a political, cultural, and economic centre. The study examined the “Beijing Paradise Supermarket Bar COVID-19 Pandemic in 2022” incident, a highly influential and illustrative public health event from the post-pandemic period, as a case study. On 9 June 2022, a cluster outbreak linked to a bar in the Chaoyang District was detected during routine screening efforts (Figure 1). The outbreak was primarily attributed to large-scale gatherings where protective measures were not adequately observed. By 10 June, the event had evolved into a widespread cluster outbreak, characterised by rapid transmission, extensive geographic impact, and a diverse composition of affected individuals (Reuters, 2022). This incident not only posed significant challenges for epidemic control within Beijing but also led to the emergence of secondary outbreaks in other regions across China, highlighting the interconnectedness of urban mobility and public health crises.

**Figure 2:**
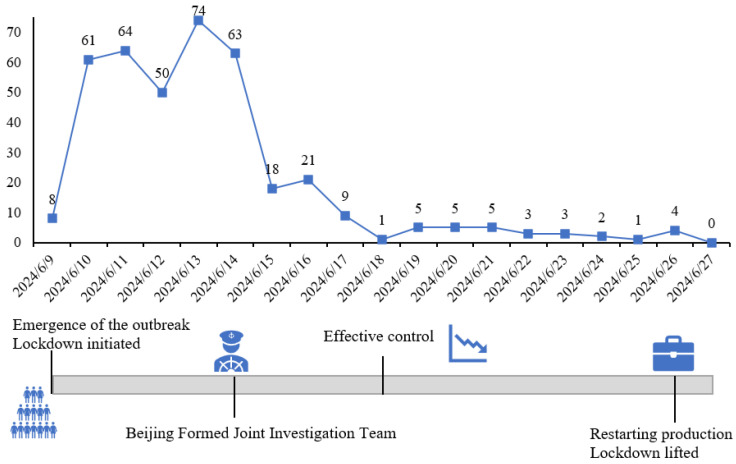
Daily new cases in Beijing and key milestones of the outbreak.

Figure 2 illustrates the key milestones and daily new case numbers for this outbreak. (1) On 9 June, a bar-related cluster outbreak emerged in Chaoyang District, prompting citywide inspections of bars, internet cafes, and similar venues in Beijing. Entertainment venues were closed, and lockdown measures were implemented; (2) On 13 June, six municipal departments formed a joint investigation team and launched a formal investigation into Paradise Supermarket Bar in accordance with the law; (3) By 18 June, the outbreak was largely under control, although sporadic and concealed sources of infection remained in certain areas; (4) On 26 June, Beijing began to gradually resume work, production, and commercial activities, with students returning to schools and lockdown measures lifted.

### 2.2. Data Access and Preprocessing

Weibo is one of the most popular social media platforms in China, often referred to as the Chinese version of Twitter (Yang et al., 2022). The platform enables users to share information, follow trending topics, evaluate public events, and express personal emotions in real time. Unlike other social media platforms that primarily focus on private interactions, Weibo is designed for open and large-scale public discourse, making it particularly suitable for analysing public sentiment and opinion trends. Its interactive features, such as reposts, comments, and likes, provide valuable indicators of information diffusion and engagement. Furthermore, Weibo played a central role in shaping public discussions during major events, including the post-COVID-19 pandemic, making it an essential platform for studying crisis-related sentiment dynamics. Taking the Beijing Paradise Supermarket Bar outbreak as an example, Weibo captured the majority of public opinion and emotional responses during the event. Analysing these data provides significant research value and meaningful insights into understanding public sentiment and opinion dynamics in crisis situations.

Weibo data from the pandemic period were used as the primary dataset for this study. To collect the data, a web crawler was employed to automatically retrieve Weibo posts containing the keywords “Beijing bar-related outbreak” and “Paradise Supermarket bar outbreak”. This approach ensured the systematic acquisition of relevant public discourse during the specified time frame. We crawled and filtered Weibo text data spanning from the onset of the public health crisis to the data collection period (9 June to 18 August 2022). The dataset included usernames, Weibo content, comment content, posting times, posting locations, number of likes, and number of comments, in alignment with the requirements of the study.

The collected data underwent a comprehensive preprocessing phase to ensure quality and relevance for subsequent analysis. Noise and irrelevant content were removed, including invalid elements such as filler words (e.g., um), repost indicators (e.g., repost), user mentions (e.g., @username), hyperlinks, and other indiscernible content. Emojis with emotional content were converted into their corresponding Chinese interpretations to enhance consistency and accuracy in computational analysis. Text segmentation was performed using the Jieba tool, and high-frequency pandemic-related terms, such as “弹窗” (epidemic pop-up window, a digital alert restricting access to certain venues or travel based on health status), were identified and incorporated into the segmentation system to optimise the results. Ultimately, a refined dataset of 7808 Weibo posts was obtained, forming a corpus of public emotions. Based on previous research, the size of this single-case dataset is sufficient to support the study’s objectives (Y. Guo et al., 2023; Ma et al., 2023).

### 2.3. Method

#### 2.3.1. Emotion Analysis

Natural language processing (NLP) is a tool for how computers can understand and process human language. Sentiment analysis is an important branch of NLP that aims to identify and extract emotional tendencies and attitudes in text. In text emotion analysis, there are two main research paths for scholars: classifying text emotion by manually building a corpus to train machine learning to construct models, such as support vector machine (Zhang et al., 2019), naive Bayes model (Abbas et al., 2020), and convolutional neural networks (Severyn & Moschitti, 2015). The other is the emotional analysis of texts based on the construction of an emotional lexicon, mainly the Hownet Sentiment Dictionary, the National Taiwan University Sentiment Dictionary, the Dalian University of Technology Internet Research (DUTIR) Sentiment Lexicon, and the Tsinghua University Positive and Negative Sentiment Dictionary. Although the lexicon method relies on the quality and capacity of emotion lexicons, it is more effective in reflecting the structural characteristics of texts and is easier to understand. Therefore, this study chose a lexicon-based approach to analyse public emotions in public health crises during the post-pandemic era. We performed emotion classification and annotation on the preprocessed data using the DUTIR Sentiment Lexicon to identify emotional valence, intensity, and type (W. Wang et al., 2024; Xu et al., 2008). Meanwhile, the valence and intensity of high-frequency new words were manually marked using synonyms, antonyms, and the Delphi method. This process contributed to the expansion of the emotion lexicon, resulting in the creation of a pandemic-specific emotion ontology, which was incorporated into subsequent analyses.

The emotional score calculation in the study was based on emotion word matching. The main process involved identifying emotion words, negation words, and degree words in a sentence, and then arranging them in the order of their occurrence. Adjacent negation or degree words were identified within this sequence, and their weights were multiplied to obtain the new weight of each emotion word. The overall emotion score of a sentence was derived by summing the emotion weights of all emotion words. This study analysed the emotions in the crawled text using a revised emotion lexicon, with results further refined through manual review.

#### 2.3.2. Spatial Autocorrelation Test

The spatial autocorrelation test is a statistical method used to measure the degree of similarity or correlation of a variable across space. It assesses whether the values of a given variable at one location are systematically related to the values of the same variable at neighbouring locations. This test helps identify spatial patterns, such as clustering or dispersion, in spatial data, and is commonly used in geographic and environmental studies to understand spatial dependencies within a dataset (Altaweel, 2017). In this study, a spatial autocorrelation test was performed to analyse the spatial patterns of public emotion heat values across different periods, evaluating the degree of spatial dependency between public emotions during the outbreak and its subsequent phases.

Both global and local indicators of spatial association exist. Global spatial autocorrelation reflects the spatial aggregation degree of public emotion heat across the nation. By analysing this under different periods, the pattern of global spatial aggregation of public emotion heat over time can be obtained. This value is measured using Moran’s *I* index, defined as follows:

(1) $$I=\frac{\sum_{i=1}^{n}\sum_{j\neq i}^{n}w_{ij}\left(x_{i}-\bar{x}\right)\left(x_{j}-\bar{x}\right)}{S^{2}\sum_{i=1}^{n}\sum_{j\neq i}^{n}w_{ij}}$$

Here, *I* indicates the global Moran’s *I* index, *n* denotes the number of all provinces in space, *x_i_* and *x_j_* are the emotional heat values in provinces *i* and *j* at period T, respectively, $\bar{x}$ is the mean emotional heat value, and *S*^2^ is the variance of the emotional heat value in all provinces in period T. *w_ij_* is the spatial weight matrix representing the neighbourhood relationship between provinces *i* and *j*. The value of *I* ranges from −1 to 1: a value greater than zero indicates similar public emotion heat values exist in the neighbourhoods; a value less than zero suggests diverse heat values in the neighbourhoods, and a value equal to zero suggests no correlation. A larger value of *I* indicates a higher degree of spatial aggregation of public emotions.

To test for spatial autocorrelation between *n* regions, the normalised statistic *Z*, based on global Moran’s *I*, can be calculated as follows:

(2) $$Z=\frac{I-E(I)}{\sqrt{\mathrm{VAR}(I)}}$$

Here, *E*(*I*) and *VAR*(*I*) are the expectation and variance of public emotion heat value, respectively. When *Z* is positive and significant, it indicates a positive spatial autocorrelation of public emotion heat, meaning that similar observations tend to be spatially agglomerated. When *Z* is negative and significant, there is a negative spatial autocorrelation and similar observations tend to be dispersed. When *Z* is zero, the observations are randomly and independently distributed.

Local spatial autocorrelation reflects the differences in the local spatial distribution of public emotion heat and explores the spatial heterogeneity of emotional heat at different stages of the public opinion circle. The local spatial autocorrelation was measured using the local Moran’s *I* index:

(3) $$I_{i}=\frac{\left(x_{i}-\bar{x}\right)\sum_{j=1}^{n}w_{ij}\left(x_{j}-\bar{x}\right)}{S^{2}}$$

Here, *I_i_* refers to local Moran’s *I*, and the rest of the parameters have the identical meaning as in Equation (1). According to the local spatial autocorrelation analysis, four types of spatial aggregation of public emotion heat exist: high–high aggregation, high–low aggregation, low–high aggregation, and low–low aggregation, which represent hotspot areas, high-value anomaly areas, low-value anomaly areas, and cold-spot areas, respectively.

The formula for the local Moran’s *I* test is as follows:

(4) $$\;Z\left(I_{i}\right)=\frac{I_{i}-E(I_{i})}{\sqrt{\mathrm{VAR}(I_{i})}}$$

Here, the variables have the same meaning as in Equation (2). When *Z*(*I_i_*) is significantly positive, it indicates that the regional units with high observed values around the region tend to be spatially agglomerated. Significantly negative values indicate that regional units with low observed values tend to spatially agglomerate.

#### 2.3.3. Content Analysis

Content analysis is a viable approach for thematising public commentary. As a widely used data analysis method, it focuses on developing categories from data while recognising the importance of understanding and analysing their contextual significance (Krippendorff, 2019). It has been increasingly applied to the study of user-generated content (UGC), which includes text, images, videos, and audio shared by users on online platforms such as social media. Quantitative content analysis emphasises counting and measurement, aiming to systematically and reproducibly quantify content based on predefined categories. In contrast, qualitative content analysis focuses on interpretation and understanding, with researchers iteratively refining themes or categories through continuous conceptualisation, data collection, analysis, and interpretation (Vespestad & Clancy, 2021; Luo, 2019). This approach enables researchers to identify patterns and categories within the data, thereby allowing for a deeper understanding of public sentiment and discourse in social media contexts.

In this study, a content analysis approach was employed to analyse thematic changes under varying public emotions. Texts associated with positive and negative emotions were selected as the raw data for coding analysis. This study conducted a content analysis of public discourse across different emotional valences to explore the categories of positive and negative emotions, their influencing factors, and the resulting public responses. Throughout the research process, it is essential for researchers to uphold a qualitative perspective. The primary concern is to ensure rigour and reliability, thereby maximising the accuracy of the findings. An agreement level of 80% is generally considered an acceptable threshold for validity (Bengtsson, 2016). Additionally, word clouds were utilised to visualise the spatial and temporal dynamics of public discourse across different periods and regions.

#### 2.3.4. Period Division of Public Emotion

Scholars have meticulously delineated the stages of crisis dissemination. For example, Steven Fink proposed a four-stage theory of crisis dissemination, including the crisis potential stage, crisis emergency stage, crisis contagion stage, and finally the crisis resolution stage, which is a cycle of the full stages of crisis dissemination (Fink, 1986). The evolutionary modes of online public opinion events mainly have a single-peak shape, double-peak type, and multi-peak type. Public emotions on social media exhibit temporal correlations with the ongoing development of the pandemic over different time periods. As the situation progresses, changes in public sentiment often reflect key milestones in the pandemic’s trajectory, such as policy updates, outbreaks, or containment measures. Therefore, in this study, we chose emotional value as an indicator to divide the research phases. While previous studies have generally divided the stages based on the development of the pandemic or the volume of Weibo texts (W. Wang et al., 2024), our approach, based on emotional value, provides a more appropriate and comprehensive framework for describing the spatiotemporal evolution of public emotions. This method offers a nuanced understanding of the dynamic shifts in public sentiment, particularly how emotions evolve in relation to specific stages of the pandemic, rather than simply correlating with the progression of cases or text frequency.

### 2.4. Proposed Methodology

**Figure 3:**
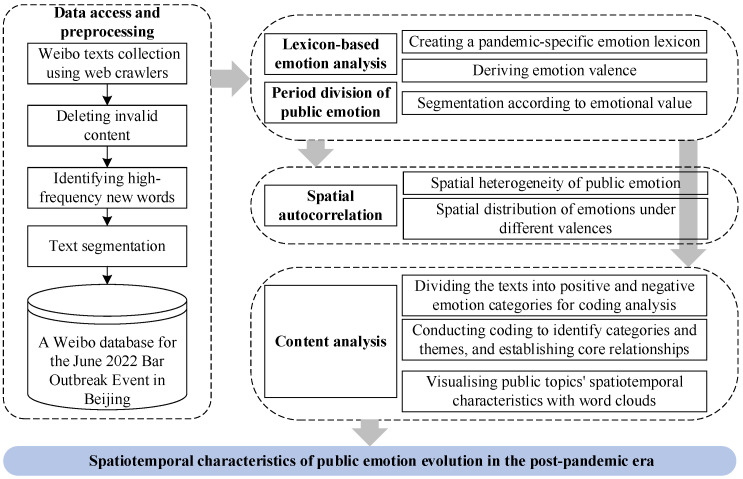
Research framework.

In this study, various analytical methods were employed to evaluate the spatiotemporal characteristics of public emotion evolution during the post-pandemic period. Figure 3 illustrates the research design, where different methods were used in parallel, analysed separately, and subsequently integrated for comprehensive insights.

The methodology of this study was organised into three main phases: (1) Data collection and pre-processing. In the first phase, web crawlers were employed to collect Weibo posts related to the bar outbreak. Following data collection, irrelevant and invalid content was removed to ensure data quality. Additionally, newly identified words were incorporated into the emotion lexicon to enhance the accuracy of emotion analysis. Word segmentation was also performed to facilitate a more precise evaluation of emotion. These steps led to the construction of a structured Weibo database, forming the foundation for subsequent analyses. (2) Emotion analysis. In the second phase, a lexicon-based emotion analysis method was employed to effectively identify the emotional polarity of the texts. Based on the calculated emotional values, the public emotion evolution cycle was segmented into distinct phases, allowing for a detailed examination of how public sentiment changed over time. (3) Spatial autocorrelation and content analysis in parallel. In the third phase, spatial autocorrelation tests were applied to examine the spatial clustering patterns of public emotions across different time periods. Additionally, the spatial distribution of emotions under different valences was analysed to uncover regional variations. Content analysis was performed in parallel, systematically coding texts with positive and negative emotional valences separately to explore the key factors influencing public emotions. To further illustrate regional thematic variations, word cloud visualisation was employed. Building upon these analytical steps, a conceptual model was developed to analyse the spatiotemporal characteristics of public emotions in the post-pandemic era.

## 3. Results

### 3.1. Spatiotemporal Characteristics of Public Emotions

#### 3.1.1. Stage Division of Public Emotions

**Figure 4:**
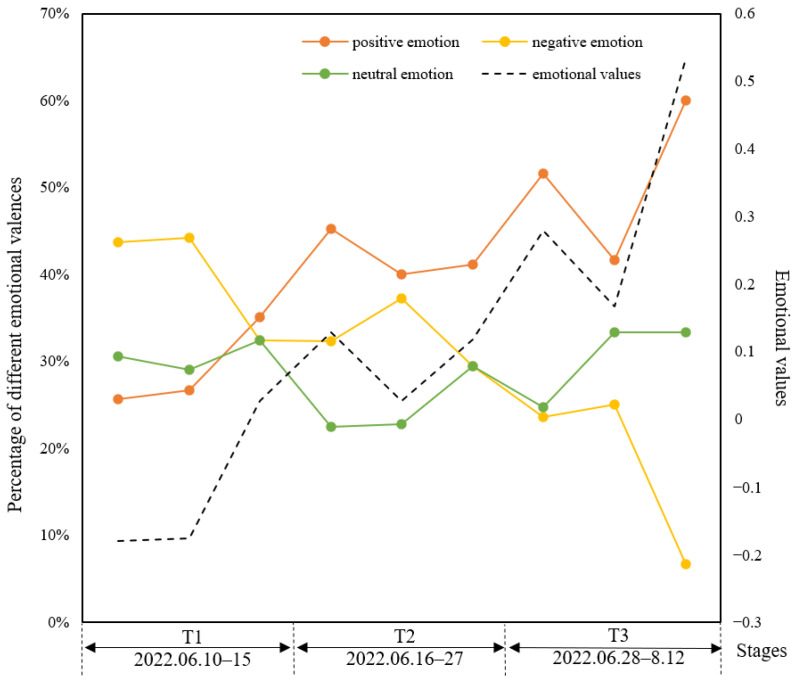
Emotion values trends and different emotional valence percentages.

Based on the crisis communication life cycle and the evolution model of online public opinion, combined with the trend of emotional valence (Figure 4), we defined the evolution pattern of this pandemic as multi-peaked, divided into three time periods: T1, T2, and T3, which correspond to the emergency stage, the contagion stage, and the resolution stage, respectively.

The analysis of textual emotional values (Figure 4) indicates an overall upward trend of emotional values over time during public health crises. In conjunction with the number of new cases (Figure 2), period T1 refers to the five days preceding the event, a critical period marked by a surge in cases followed by initial containment efforts. Emotional values were below zero during period T1, implying more negative emotions. Period T2 corresponds to the period when the number of new cases steadily declined until no further cases were reported. During this phase, emotional values exhibited some fluctuations but remained predominantly positive. Period T3 represents the post-event recovery stage, characterised by a significant increase in emotional values, with positive emotions accounting for more than half of the total. From period T2 onwards, emotional values fluctuated above zero, signifying a general increase in positive emotions after the first crisis period. Nonetheless, emotional values experienced ups and downs owing to the fluctuating trajectory of the pandemic. Throughout the crisis, neutral emotions hovered around 30% of the total. Negative emotions showed a continuous decline in volatility while positive emotions were on an upward trend.

#### 3.1.2. Spatial Heterogeneity Analysis of Public Emotion Across Stages

In the spatial autocorrelation analysis, log function standardisation of the raw data was used to eliminate differences of orders of magnitude. In the construction of the spatial weight matrix, owing to the large differences in the area of China’s provincial administrative units and the existence of non-connected provinces, the *k*-nearest neighbour was used to construct the spatial weight matrix. Given a positive integer *k*, the weight of the *k* nearest provinces to the target province was one, and the rest of the provinces were zero. Where a value of nine was chosen for *k*, the significance level of the spatial autocorrelation analysis of the public emotion heat was higher, at which point Moran’s *I* = 0.165 > 0, *p*-value = 0.008 < 0.05, indicating that the spatial distribution of public emotion heat in public health crises has a certain degree of aggregation, and the confidence level of the results of this analysis is over 90%.

The results of the global spatial autocorrelation analysis of the public emotion heat are shown in Table 1. All Moran’s *I* values were greater than zero, indicating that the public emotion heat followed a pattern of spatial aggregation. The degree of aggregation initially strengthened and then weakened over time, with the weakest aggregation occurring at the third stage. Meanwhile, the *p*-value for each period was less than 0.05, and the *Z*-score was greater than 1.65, indicating a confidence level above 90% (Briz-Redón & Serrano-Aroca, 2020).

**Figure 5:**
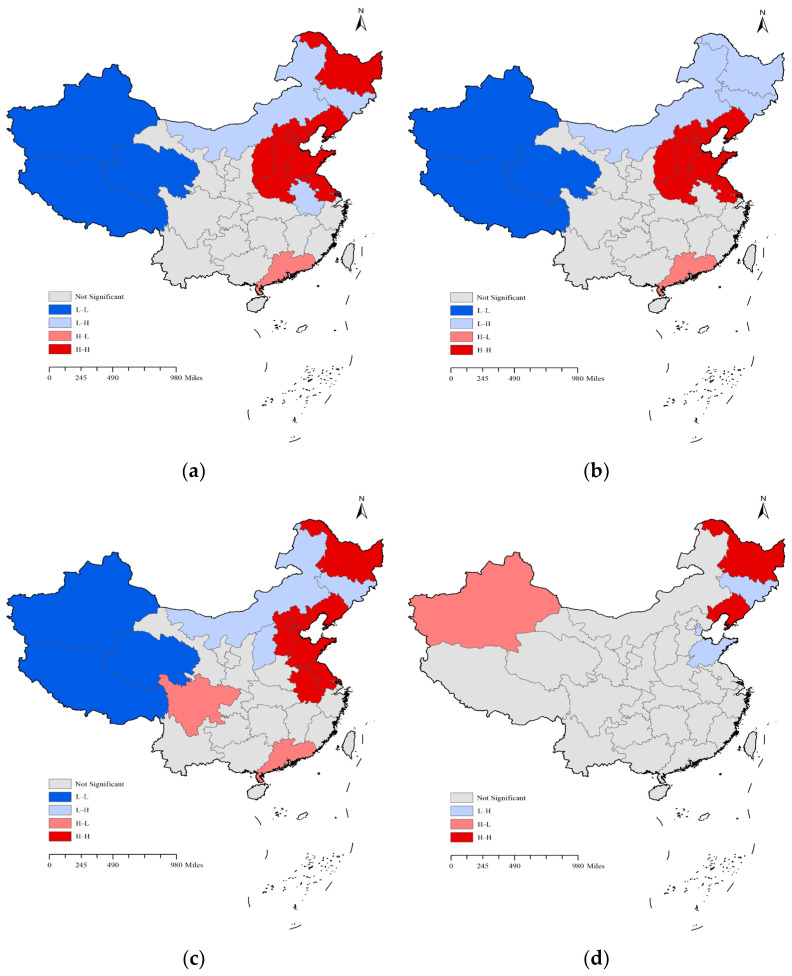
Maps of local aggregation types by stages. (**a**) T stage; (**b**) T1 stage; (**c**) T2 stage; (**d**) T3 stage.

The results of the local spatial autocorrelation analysis are presented in Table 2 and Figure 5. Throughout the crisis, the number of high–high aggregation areas was high, signifying the presence of numerous public emotion hotspots. These were primarily concentrated in Beijing, Tianjin, Hebei, Shanxi, and Liaoning, and other such regions. High–high aggregation areas were mainly in and around the outbreak areas, suggesting frequent movement and inter-provincial commuting, which heightened sensitivity to emergency-induced emotions. Furthermore, areas such as Liaoning, which were recently suffering from public health crises, could also cause emotional resonance and evoke public emotions at the time of the event. High–low and low–high aggregation areas suggested the heterogeneity of public emotion hotspots nationwide. For instance, a diagnosed individual visiting Guangdong, a region distant from the event, caused a temporary surge in online public emotions. Low–high aggregation areas, which mirrored high–high aggregation areas, reflected local low values of public emotion heat, primarily in Inner Mongolia and Jilin. The limited number of low–low aggregation regions suggested the presence of spatial cold spots, such as Xinjiang and Tibet, though these were relatively few. In addition, there were more regions with insignificant spatial aggregation than significant regions, indicating the nationwide prevalence of public emotion hotspots during all periods, not just a pandemic in parts of the country. Comparing the changes in the number of different aggregation types across periods, high–high aggregation maintained high and constant values during the T1 and T2 periods of public health crises and reached its lowest point in the T3 period. This trend mirrored that of the global Moran’s *I*, suggesting that the spatial aggregation intensity of public emotion heat values corresponded with the number of spatial hotspot regions.

#### 3.1.3. Spatial Distribution of Different Emotional Valences Across Stages

**Figure 6:**
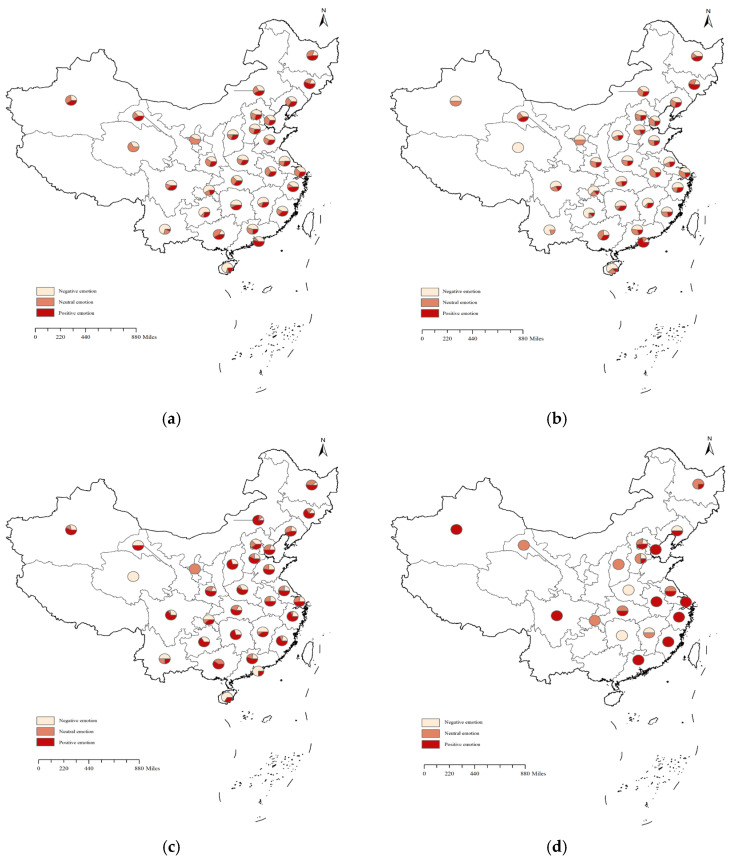
Maps of different emotional valence by stages. (**a**) T stage; (**b**) T1 stage; (**c**) T2 stage; (**d**) T3 stage.

After analysing the emotional valence of the emotional texts from different regions, the spatial distribution of the emotional valence in public health crises over time was obtained using three social-emotional dissemination stages as time intervals (Figure 6). It is clear from the figure that, firstly, in most areas affected by public health crises, negative social emotion dominated in the T1 period, and positive social emotion gradually emerged in the T2 period, until social emotion gradually regained its calmness and positive social emotion dominated in the T3 period. Secondly, in the later periods of public health crises, the expression of emotions in provinces other than the areas where the event occurred gradually became homogenous and no longer resonated with a wide range of social emotions, for example, in the T3 period of the crisis, while Beijing still expressed a wide range of social emotions, social emotions in other provinces were only sporadically expressed and individual emotions were positive.

### 3.2. Spatiotemporal Characteristics of Polarised Emotional Topics

#### 3.2.1. Topic Clustering and Influencing Factors Under Polarised Emotions

Previous studies on the psychological impact of the COVID-19 crisis on the public were conducted within the framework of the Stimulus-Organism-Response (S-O-R) theory (Pandita et al., 2021), which posits that external stimuli influence emotional responses, thereby shaping behavioural reactions. Accordingly, this study adopted an external stimulus–positive/negative emotion–behavioural response framework to conduct a content analysis of public discourse across different emotional valences. The analysis followed a structured methodology. First, the texts were classified as positive or negative based on emotional valence. Next, the data were familiarised, and thematic patterns of public discourse under the two different emotional valences were analysed. This was carried out through manual coding using NVivo 12 software to identify initial codes. Subsequently, the relationships between the initial codes were examined, and similar codes were merged to generate formal codes, which were then refined into main themes. To ensure inter-coder reliability, two independent coders were employed, and coding consistency checks were performed.

Through the coding process, we identified 90 sub-categories. Based on the meanings conveyed by these sub-categories and their interrelationships, we further synthesised 24 main-categories. Specifically, 11 main-categories emerged from positive emotion texts, including strengthening pandemic measures, hopefulness, supporting pandemic policies, etc., whereas 13 main-categories were derived from negative emotion texts, such as confused notifications, criticise negligent individuals, indifference, etc. Based on the S-O-R theory, these main-categories were further refined into themes, resulting in a total of nine themes-four identified in the positive emotion texts and five in the negative emotion texts. The positive emotion themes include pandemic improvement, implementing policies, positive public emotions, and recommendations for action. The negative emotion themes include severe pandemics, prevention loopholes, restrictions on life and rights, negative public emotions, criticism and accountability. A detailed classification is presented in Table 3.

**Figure 7:**
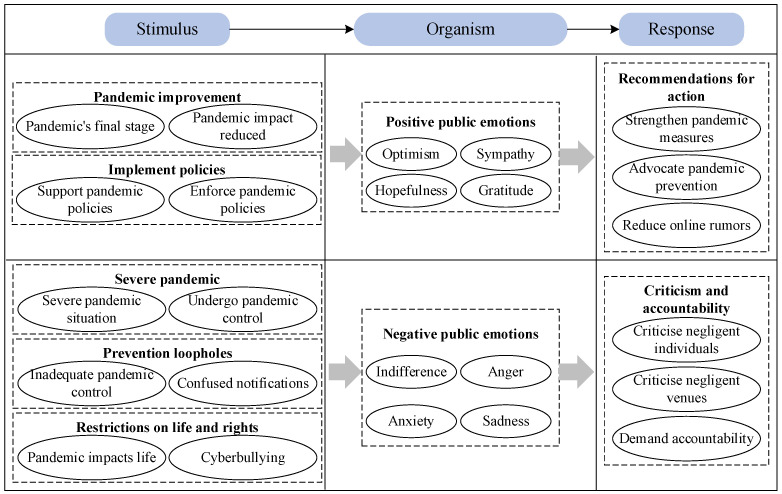
Typical themes and main-categories relationship structure.

The core relational structures under different emotional valences are illustrated in Figure 7. As shown in Figure 7, the subjective factors influencing positive public emotion mainly stem from improvements in the pandemic situation and the implementation of pandemic control policies. Positive public emotions include hopefulness, gratitude, optimism, and sympathy. The resulting online behavioural responses primarily involve making recommendations for future pandemic prevention, including calls for the government to strengthen pandemic prevention measures, individuals actively supporting pandemic control, and joint efforts to resist online rumours. The subjective factors influencing negative public emotion primarily arise from the severity of the pandemic, insufficient pandemic control, and restrictions on life and rights. Negative public emotions include indifference, anger, anxiety, and sadness. The corresponding online behavioural responses mainly involve criticism and accountability, including criticism of individuals and places involved in inadequate pandemic control and call for holding these behaviours accountable.

#### 3.2.2. Spatiotemporal Evolution Characteristics of Topic Clustering

**Figure 8:**
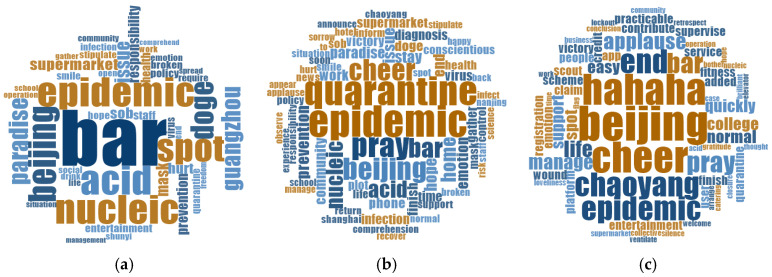
Topic word cloud maps by stages. (**a**) T1 stage; (**b**) T2 stage; (**c**) T3 stage.

To further explore the spatial and temporal dynamics of public discourse, we used word clouds to visualise the distribution of themes and examined regional differences through geotagged content. Focusing on residents’ concerns during the three periods of public emotion evolution, topic word cloud maps were drawn during the post-pandemic era (Figure 8). Overall, the residents continued to focus on keywords such as “bar”, “Beijing”, and “pandemic”, and there was a change of topics from “the current situation of the pandemic” in the T1 period to “active prevention and protection” in the T2 period to “positive pray and blessings” in the T3 period. In the T1 period, residents’ focus on the source and spread of the pandemic was accompanied by negative emotions, as evidenced by the high frequency of words like “bar”, “Beijing”, “nucleic acid”, “pandemic”, “mask”, etc. indicating their concern about the pandemic’s origins and the preventive measures taken. High-frequency words such as “hurt”, “sob”, and “doge” also reflected the residents’ negative emotions at this time. During the T2 period, words like “quarantine”, “pandemic prevention”, “pray”, “cheer”, etc. suggested a shift towards current pandemic prevention measures, with emotions gradually developing in a positive direction. In the T3 phase, terms like “end”, “normal”, “hahaha”, “applause”, “pray”, “cheer”, etc. reflected the end of the public health crises and the rise of positive social emotions.

**Figure 9:**
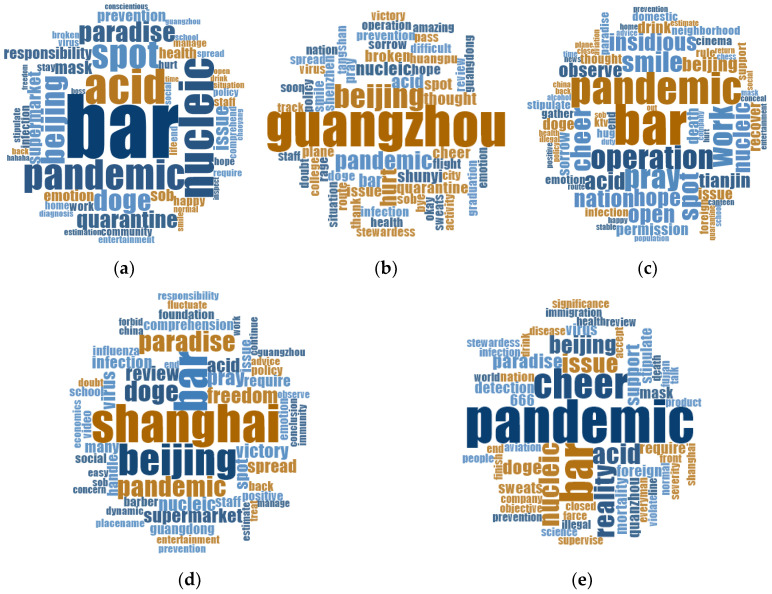
Topic word cloud maps for five provinces in China. (**a**) Beijing; (**b**) Guangdong; (**c**) Tianjin; (**d**) Shanghai; (**e**) Fujian.

Based on the overall spatial distribution of the public emotion heatmaps, five illustrative provinces were selected to explore the topic clustering of residents in different geographical areas under the same public health crisis (Figure 9). These included Beijing (where the public health crisis occurred), Guangdong (where the infected visited spatially and temporally), Tianjin (a geographically close area), Shanghai (a region that had recently experienced a similar event), and Fujian (a region that was geographically distant and less affected). Firstly, in Beijing, the location of the outbreak, the residents were concerned about the outbreak spots such as “bars” and “paradise”, as well as the current state of the pandemic control such as the “nucleic acid”, and “pandemic”. At this time, public emotion was mostly negative, as evidenced by words like “sob” and “hurt”. Secondly, in Guangdong, where the infected visited spatially and temporally, residents were concerned about the spot where the pandemic had spread, such as “Guangzhou”, and the current state of pandemic control, increasing their anger towards the infected who had visited spatially and temporally, demonstrated by the use of the word “rage”. In addition, the residents in Tianjin and Shanghai not only showed concern about the development of the pandemic, pandemic prevention measures, and negative emotions, but also about public health crises triggered by similar causes in public places like “ktv”, “cinema”, and “barber”. There were also positive public emotions based on “pray” and “victory”. As a region that recently experienced similar incidents, the residents of Shanghai expressed concerns about their freedom rights. Finally, residents in the more distant and less-affected regions of Fujian showed concern about the development of the pandemic, prevention measures, and expression of different public emotions.

## 4. Discussion

This study investigated the spatiotemporal characteristics of public emotions during post-pandemic public health crises, revealing critical insights into the distribution, evolution, and influencing factors of public emotion.

The evolution of public emotions in post-pandemic public health crises exhibits a “water wave explosion” effect over time (Liu, 2017), often triggering a nationwide public response in a short span. With the rapid advancement of digital communication technologies, the public emotional cycle has become significantly shorter, with a much lower threshold for emotional reactions. Crisis-related emotions now erupt suddenly and undergo several waves of fluctuation as official announcements bring the issue back into public discourse. Unlike traditional crises, where emotions gradually build up, public sentiment now spreads almost instantaneously, bypassing the incubation period and entering the emergency phase directly. This shift renders Steven Fink’s four-stage crisis dissemination model less applicable, as the potential crisis phase in public health emergencies is no longer distinct, and the emergent crisis stage follows the event almost immediately (Hu, 2022).

The overall spatiotemporal characteristics of public emotions during the post-pandemic era demonstrated a spatial clustering distribution. The degree of clustering initially strengthened but gradually weakened as the development of public opinion events progressed. As the pandemic worsened or improved, residents’ emotions tended to shift towards increasing negativity or positivity. Throughout the three stages of public health crisis development, positive public emotions were greater than negative ones in the latter two time periods. Spatially, more than 74% of areas exhibited a predominance of positive emotions following the initial stage, facilitated by proactive government measures in prevention, control, guidance, and intervention. This observation aligns with prior studies that have highlighted the distinct evolutionary paths of social emotions as events unfold (Q. Li et al., 2020; Hu, 2022; Jia et al., 2025). In the early stages of a public health crisis, residents often experience heightened panic due to limited information, the worsening pandemic situation, and the rapid transmission of news (Lwin et al., 2020). As the pandemic situation improves or as the government implements effective prevention and control measures, the negative emotions of residents tend to subside (Zhang et al., 2020). Public emotions during post-pandemic public health crises affected the entire nation rather than being confined to specific regions. The spatiotemporal clustering distribution exhibited geographical variability, featuring spatial hotspots, coldspots, high–low cluster areas, and low–high cluster areas of public emotions. Spatial hotspots were primarily located in and around areas directly impacted by the events (Shen et al., 2021), as well as regions recently affected by such crises. High–low aggregation areas were mainly situated in locations visited by infected individuals, both spatially and temporally (Shen et al., 2021). The distribution of low–high and low–low aggregation areas may be influenced by factors such as population density, economic development, and geographical location.

In the post-pandemic era, public positive emotions can be categorised into optimism, sympathy, hopefulness, and gratitude. These emotions are influenced by factors such as improvements in the pandemic situation and the implementation of effective policies. In turn, they foster constructive behavioural outcomes, such as proposing measures to mitigate the impact of the pandemic. Negative public emotions include indifference, anger, anxiety, and sadness, driven by factors such as the severity of the pandemic, loopholes in prevention measures, and restrictions on daily life and individual rights. Unlike during the peak of the pandemic, when public concern was primarily centred on COVID-19 and the effectiveness of preventive measures, negative emotions in the post-pandemic period have increasingly shifted towards broader societal issues, such as restrictions on life rights and online rumours. Additionally, having experienced multiple waves of outbreaks across different locations, the public has developed a sense of indifference and desensitisation that was not evident during the initial phases of the pandemic. As a result, these negative external stimuli have led to more adverse behavioural outcomes, including public criticism of individuals or venues perceived to have mishandled prevention efforts and demands for accountability. This highlights the public’s growing emotional expectation for stronger governmental enforcement to safeguard their safety, health, and the fair implementation of policies.

Public concern regarding public health crises during the post-pandemic period was diverse in both temporal and spatial dimensions. From a temporal perspective, studies have shown that the number of individuals infected by the pandemic becomes the most critical public concern in social networks during a public health crisis (Cai et al., 2021). In this study, while residents’ focus at different stages remained primarily on the progression of the pandemic, the intensity of discussions about the outbreak itself diminished over time and gradually shifted to other aspects. These included prevention and control measures, expectations for the end of the outbreak, and expressions of encouragement (Q. Li et al., 2020). This pattern reflects the temporal dynamics of public emotion in relation to evolving topics. Spatially, consistent with previous studies on the initial outbreak of COVID-19 in China, where residents were primarily concerned about the progression of the pandemic and exhibited high-stress levels, while other regions focused on pandemic prevention and control (Zhuang et al., 2021), this study also demonstrated notable geographical heterogeneity. Residents in Beijing were more concerned with the development of the pandemic and typically expressed more negative emotions compared to those in other provinces. However, the study also revealed that regions across the country, which experienced spatial and temporal visits from infected individuals, exhibited similar concerns to Beijing, primarily focused on the pandemic’s progression. In some regions, concerns are also centred on public health crises of a similar nature, further reinforcing the idea that similar event topics evoke similar public emotions (An et al., 2022). Furthermore, some studies suggest that negative emotions exert a stronger influence on public opinion and resonance than positive emotions (G. Wang et al., 2019), underscoring the importance of closely monitoring negative public emotions during such crises. The rapid decline of negative emotions does not imply their immediate disappearance upon the resolution of the crisis. For instance, post-crisis trauma may linger among residents (Cao et al., 2021) until the overall impact of the event on collective emotions has fully dissipated.

## 5. Conclusions

This study focused on the spatiotemporal characteristics of public emotion evolution during the post-pandemic era, adopting various analytical methods to analyse the temporal and spatial dimensions of social media data from a typical case in Beijing. The key findings of this study are as follows:

First, in the post-pandemic era, public health crises trigger a faster and more volatile evolution cycle, consisting of the emergency stage, the contagion stage, and the resolution stage. Second, the spatial distribution of public emotions exhibits significant clustering, with the intensity of clustering first increasing and then gradually decreasing. The spatiotemporal distribution patterns include spatial hotspots, cold spots, high–low clustering areas, and low–high clustering areas. Third, the interplay between influencing factors, public emotions, and online behaviours reveals that positive emotions (e.g., hopefulness, gratitude, optimism) are driven by pandemic improvements and policy implementation, encouraging supportive behaviours such as advocating for prevention measures and resisting misinformation, while negative emotions (e.g., anger, anxiety, sadness) stem from severe outbreaks, insufficient controls, and restrictions on freedoms, leading to criticism and demands for accountability. Finally, in the post-pandemic era, the public focus shifted from the outbreak to control measures and, ultimately, to optimism about its end. While affected areas tracked the pandemic’s progression, other regions prioritised prevention and control efforts.

The results of this study have important implications for public emotional management and mental health in the post-pandemic era, as well as for the stability of urban social order. On a theoretical level, the research developed a conceptual model to mine social media data, extending and enriching the theory and methodology of public emotion research. Practically, the empirical analysis identifies the spatiotemporal aggregation of public emotions in Beijing—the core area of public health crises—and demonstrates its influence on public emotions across the country. This provides a valuable reference for psychological health management during public health crises in large cities. Ultimately, the findings lay the foundation for developing an integrated framework for public emotion governance. By addressing spatial and temporal variations in public emotions and leveraging geospatial analytics, authorities can transform public sentiment from a source of social risk into a catalyst for societal stability and cohesion.

Based on the findings of this study, several recommendations can be made to enhance the management of public emotions during post-pandemic public health crises: (1) Proactive emotional monitoring. Given the spatiotemporal clustering of public emotions, it is crucial for authorities to implement continuous monitoring systems that can quickly identify emotional hotspots. Leveraging big data and geospatial technologies will allow for real-time tracking of public sentiment and the early detection of potential emotional crises. (2) Targeted psychological interventions. The study highlighted the variability in emotional responses across different regions. Therefore, targeted psychological interventions should be designed to address the specific emotional needs of distinct geographic areas, particularly those showing concentrated negative sentiment. (3) Timely public communication. The rapid spread and quick dissipation of emotions in the digital age underscore the importance of effective public communication. Authorities should disseminate clear, transparent, and timely information to reduce panic and confusion, particularly in the early stages of a public health crisis. (4) Post-crisis emotional support. Negative emotions may persist even after the crisis has subsided, evolving into more extreme sentiments if not adequately addressed. Post-crisis emotional support should be provided to residents, focusing on recovery and rehabilitation, including mental health services and community rebuilding initiatives. (5) Encouraging positive public engagement. Authorities should promote the dissemination of positive, constructive content through trusted community members to counteract negative emotional responses. This will help foster resilience, strengthen social bonds, and facilitate the transition from negative to positive emotions. (6) Policy transparency and fairness. Public concern regarding the fairness of policy enforcement and the economic impacts of pandemic control measures should be addressed through transparent communication and equitable policy implementation. Addressing these concerns will help to restore trust in governmental actions and prevent public resentment.

The study has several limitations. Firstly, the data sample exhibits an age imbalance. While Weibo, a major social media platform in China, covers most netizens, it overlooks vulnerable groups such as the elderly and children, who are less likely to express opinions online. Secondly, the sentiment analysis method’s accuracy depends on the completeness of the sentiment lexicon, which adds to the time and effort required. Finally, although single-case studies have limited external validity (e.g., our findings may not be directly applicable to post-pandemic events in other countries), they offer profound insights into specific phenomena by uncovering dynamic processes within complex contexts, which remains a valuable contribution to research.

Future research could incorporate more advanced and precise methods, such as large language models and deep learning, to enhance and complement fine-grained sentiment analysis. Moreover, future studies could focus on the spatial and temporal distribution and propagation of a specific emotion, such as panic, during the later stages of a pandemic for more in-depth exploration. Furthermore, as short-video platforms such as TikTok increasingly become spaces for public discourse, conducting content analysis on video and audio data could offer valuable insights into public emotional responses and topic distributions in the context of sudden public crises, presenting a promising avenue for future research.

## Figures and Tables

**Table 1:** Global Moran’s *I* and test values for the public emotion heat.

Stage	Moran’s *I*	*p*-Value	*Z*-Score
T	0.165	0.008	3.2458
T1	0.168	0.005	3.2662
T2	0.198	0.004	3.8896
T3	0.059	0.025	2.1183

**Table 2:** Statistics on local aggregation types of public emotion heat.

Stage	Aggregation Types				
	High–High (H–H)	Low–Low (L–L)	High–Low (H–L)	Low–High (L–H)	Not Significant
T	9	3	1	3	18
T1	8	3	1	3	19
T2	8	3	2	3	18
T3	2	0	1	3	28

**Table 3:** Analysis results of theme, main-category, and sub-category.

Theme	Main-Category	Sub-Category
Positive emotion texts		
Implement policies	Support pandemic policies	Dynamic zero-COVID; Summarise experiences
	Enforce pandemic policies	Strict prevention; Effective accountability; High efficiency; Proactive prevention; Solve problems
Pandemic improvement	Pandemic’s final stage	Zeroing cases; Epidemic under control
	Pandemic impact reduced	Reduced virus virulence; Less impact on life
Positive public emotions	Hopefulness	Good health; End quarantine; End pandemic; Return to normal; Government pandemic prevention
	Gratitude	Medical staff; Pandemic cooperators
	Optimism	Pandemic situation; Personal status; Non-close contact
	Sympathy	Encouragement; Need for entertainment; Need for workplace reopening
Recommendations for action	Strengthen pandemic measures	Actively trace sources; Strengthen management; Optimise Health Code; Clarify responsibilities; Identify loopholes; Postpone indoor venue reopening
	Advocate pandemic prevention	Timely nucleic acid tests; Avoid gatherings; Adjust mindset; Take precautions; Cooperate with tracing; Follow pandemic rules
	Reduce online rumours	Do not believe or spread rumours; Reduce inappropriate speech
Negative emotion texts		
Prevention loopholes	Confused notifications	Unclear; Incomplete; Unreliable; Incomprehensible
	Inadequate pandemic control	Popup vulnerabilities; Few testing sites; Unclear source; Overzealous prevention; Spread control difficulty
Restrictions on life and rights	Cyberbullying	Discrimination; Stigmatisation; Privacy invasion; Inappropriate remarks
	Pandemic impacts life	Movement restrictions; School reopening delay; Work resumption delay; Plan disruption; Prevention fatigue; Other treatments deferred
Severe pandemic	Severe pandemic situation	Rapid outbreak; Widespread; New cases; Similar outbreaks
	Undergo pandemic control	Popup; Lockdown; Contact tracing; Symptomatic
Negative public emotions	Indifference	Unbeatable pandemic; Recurring outbreaks; Life numbness; Frustration with life; Avoid pandemic info; Loss of hope
	Anger	Prevention efforts wasted; Rule violation; Law-abiders pay
	Anxiety	Recurring pandemic; Worsening pandemic; Long-term pandemic living
	Sadness	Infected; Bitterness; Speechless
Criticism and accountability	Demand accountability	Individual negligence; Venue negligence
	Criticise negligent individuals	Gather; Roam; Refuse testing; Conceal information; No masks
	Criticise negligent venues	No temperature check; No Health Code check; No crowd control

## Data Availability

The datasets presented in this article are not readily available because the data are part of an ongoing study. Requests to access the datasets should be directed to the corresponding author (chenyan@bit.edu.cn).
